# The Treatment of Severe Periodontitis Using a Local Antiseptic Desiccant and Subgingival Mechanical Instrumentation: A Pilot Study

**DOI:** 10.3390/jcm12134286

**Published:** 2023-06-26

**Authors:** Andrada Soancă, Daniel Corneliu Leucuța, Alexandra Roman, Andreea Ciurea, Marius Negucioiu, Laurențiu Cătălin Pascu, Andrei Picoș, Ada Gabriela Delean, Iulia Cristina Micu, Aurel Popa Wagner, Darian Rusu

**Affiliations:** 1 Department of Periodontology, Faculty of Dental Medicine, Iuliu Hatieganu University of Medicine and Pharmacy Cluj-Napoca, Victor Babes St., No. 15, 400012 Cluj-Napoca, Romania; andrapopovici@gmail.com (A.S.); veve_alexandra@yahoo.com (A.R.); i.cristina.micu@gmail.com (I.C.M.); 2 Department of Medical Informatics and Biostatistics, Iuliu Hatieganu University of Medicine and Pharmacy Cluj-Napoca, Louis Pasteur St., No. 6, 400349 Cluj-Napoca, Romania; dleucuta@umfcluj.ro; 3 Department of Prosthodontics, Faculty of Dental Medicine, Iuliu Hatieganu University of Medicine and Pharmacy Cluj-Napoca, Clinicilor St., No. 32, 400006 Cluj-Napoca, Romania; sica5319@yahoo.de (M.N.); laurentiupascu@yahoo.com (L.C.P.); 4 Department of Prevention in Dental Medicine, Faculty of Dental Medicine, Iuliu Hatieganu University of Medicine and Pharmacy Cluj-Napoca, Avram Iancu St., No. 31, 400347 Cluj-Napoca, Romania; andrei.picos@umfcluj.ro; 5 Department of Cariology, Endodontics and Oral Pathology, Faculty of Dental Medicine, Iuliu Hatieganu University of Medicine and Pharmacy, Motilor St., No. 33, 400001 Cluj-Napoca, Romania; 6 Vascular Neurology and Dementia Center, University of Medicine, Essen, Hufeland St., No. 55, 45122 Essen, Germany; aurel.popa-wagner@geriatrics-healthyageing.com; 7 Experimental Research Center in Normal and Pathological Aging (ARES), University of Medicine and Pharmacy Craiova, 200349 Craiova, Romania; 8 Department of Periodontology, Anton Sculean Research Center of Periodontal and Peri-Implant Diseases, Faculty of Dental Medicine, Victor Babes University of Medicine and Pharmacy Timisoara, Bulevardul Revolutiei din 1989, No. 9, 300230 Timisoara, Romania; rusu.darian@umft.ro

**Keywords:** anti-infective agents, HYBENX, periodontitis, periodontal pocket, pilot study

## Abstract

This randomized, split-mouth, controlled clinical study assessed the additional clinical benefits of a local desiccant antimicrobial agent (HY) combined with subgingival mechanical instrumentation (SRP) vs. SRP alone in treating severe periodontitis. Patients with stages III and IV periodontitis received full-mouth periodontal examinations at baseline and after a three-month follow-up. Two randomly selected hemiarches in each periodontitis patient were treated with SRP plus HY and were included in the test group, while the other two hemiarches received only SRP and were included in the control group. In thirty patients, the analyses of the evolution of the periodontal parameters over time showed statistically significant mean differences for the probing depths and clinical attachment level values resulting from all the examined sites, as well as from the interproximal sites (*p* < 0.001) in both the test and control groups. The intergroup comparisons of the same four parameters showed no significant differences (*p* = 0.322, *p* = 0.36, *p* = 0.516, and *p* = 0.509, respectively). Based on these study results, no additional benefits were obtained after HY subgingival applications.

## 1. Introduction

Severe periodontitis is a highly prevalent chronic human disease, affecting approximately 11% of the global population [1,2]. This condition causes significant local oral alterations, impairs the quality of life, and negatively impacts systemic health [3]. Given this holistic perspective, the increased interest of clinicians and researchers in contributing to improving periodontitis therapy seems justified. The current understanding of periodontitis pathogenesis is supported by polymicrobial synergy and dysbiosis theories, stating that an increased polymicrobial community leads to subgingival dysbiosis and local immune disruption in susceptible individuals [4,5]. An excessive subgingival microbial burden generates local inflammation, altering the environmental conditions and promoting the selective outgrowth of pathobionts at the expense of “inadaptable” bacterial species, resulting in a dysbiotic imbalance in the subgingival areas [6,7]. In periodontitis, the subgingival biofilm exhibits remarkable stability and resilience, making the spontaneous return to a health-associated microbiota highly unlikely [8]. External interventions, such as subgingival mechanical instrumentation, are required to disrupt the subgingival biofilm, disperse microorganisms, alter their gene expression, increase their vulnerability [9,10], and by this means, improve clinical parameters or even achieve periodontal stability [11].

The subgingival mechanical instrumentation (SRP) in periodontitis cases, particularly in severe forms, is sometimes insufficient for achieving periodontal stability due to the inability to reach deep periodontal pockets or anatomically inaccessible sites [12], which prevents the closure of periodontal pockets and local healing. Residual pockets after non-surgical therapy are incompatible with periodontal health by maintaining a favorable subgingival environment for the persistence of a dysbiotic biofilm and inflammation, causing further periodontal destruction [13,14].

The use of locally delivered adjunctive subgingival antimicrobial products in the second phase of periodontal therapy seems to be a necessary therapeutical approach, since residual infection and inflammation need more advanced adjunctive therapeutical aids in order to achieve the desired treatment outcomes. Currently, no generally applicable drug or approach is recommended by the scientific literature to ameliorate periodontal healing following the second-phase therapy [11]. Moreover, some adjunctive locally delivered antibiotics associated with SRP provide only modest clinical benefits in terms of periodontal pocket reduction compared to SRP alone (weighted mean difference less than 0.5 mm) [11]. Thus, further research is needed to evaluate the efficacy of new adjunctive substances or products used as local adjunctive agents in the second phase of periodontitis treatment [15]. 

A commercial antimicrobial and desiccant product containing sulphonic/sulphuric acids, HYBENX^®^ (HY), Epien Medical, St. Paul, MN, USA [16], initially used to protect oral ulcerative lesions [17], has been recently recommended as a potential adjunctive drug to treat periodontitis and peri-implantitis lesions. However, there is scarce information on its clinical efficacy or action in periodontitis therapy [18]. HY has desiccation and cauterization effects and can denature and coagulate tissue debris, forming a protective coating over ulcerated areas [17,19,20]. 

HY exerts an antimicrobial effect against periodontal pathogens, particularly those belonging to the red and orange complex, and reduces periodontitis-associated inflammatory mediators, as shown in various previously conducted clinical trials and case reports [21,22,23,24]. One clinical study reported at follow-up a significant reduction in the levels of *Aggregatibacter actinomycetemcomitans*; *Porphyromonas gingivalis*; *Tannerella forsythia*; *Treponema denticola*; *Campylobacter rectus*; *Eubacterium nodatum*; *Fusobacterium nucleatum*; *Fusobacterium periodonticum*; *Peptostreptococcus (Micromonas) micros*; *Prevotella intermedia Capnocytophaga* sp. *(gingivalis, ochracea*, and *sputigena)*; and *Eikenella corrodens* after one single application of HY in a periodontitis case, while a second application of HY in combination with SRP determined a bacterial DNA reduction to a level below the DNA detection limit [21]. A case series indicated a marked reduction of the total bacterial load and of the red complex bacteria immediately after peri-implantitis treatment consisting of HY in conjunction with non-surgical or surgical treatment. However, at three months follow-up, an increase in the total bacterial load was seen [23]. Isola et al. (2018), in a split-mouth randomized clinical trial (RCT) aiming to compare the combination of SRP and HY with SRP alone, reported in favor of the combined treatment approach, a more significant decrease in the proportion of *Fusobacterium nucleatum*, *Fusobacterium polymporphum*, *Fusobacterium periodonticum*, *Prevotella intermedia*, and in all bacteria of the red complex after SRP plus HY [22]. Another RTC also found a more significant reduction of the anaerobic bacterial load following the combined treatment (HY and ultrasonic instrumentation) as compared to mechanical instrumentation alone, but at three months, no significant difference in the anaerobic bacterial burden was found between the two treatment groups [24]. HY has been reported to also significantly decrease the levels of IL-1β, IL-6, IL-10, MMP-8, and TNF-α in gingival crevicular fluid [21,22].

Although this antimicrobial, desiccant product was not considered as a therapeutical means by the recent practical guidelines [11], it has some interesting properties that deserve further analysis.

In this context, this study aimed to assess the potential additional clinical benefits of a commercial desiccant antimicrobial agent (HY) in combination with SRP vs. SRP alone for the treatment of severe periodontitis cases during a three-month interval. The following null hypothesis was tested: after three months, no differences in the modifications of the clinical parameters between the two therapeutic regimens can be found. 

## 2. Materials and Methods

### 2.1. Study Design

A pilot split-mouth, randomized, controlled clinical trial (RCT) was conducted at the Periodontology Department of Iuliu Hațieganu University of Medicine and Pharmacy/Ambulatory Care Unit of the County Emergency Hospital Cluj-Napoca between 2019 and 2021. This study was performed following, and according to the guidelines provided by the Declaration of Helsinki on experiments involving human subjects after receiving ethical approval from the Ethics Committees of both Institutions (No. 80/1.02.2018, No. 10539/B/4.05.2018, and No. 24211/B/25.10.2018). According to the institutional requirements, the registration of the study in the public trials registry was not required. Each participant was informed about the protocol and possible risks of the study and provided written informed consent before enrolling in the study. This trial was reported based on the CONSORT guidelines [25].

The inclusion criteria were •stage III or IV periodontitis [26,27], •age >18 years, •good general health, •a minimum of five natural teeth per quadrant, and •a minimum of two sites on different teeth with a pocket probing depth (PD) > 4 mm. The exclusion criteria were •periodontal or antibiotic therapy during the last six months, •pregnancy, •severe systemic conditions, •previous or current radiation or immunosuppressive therapy, and •use of antimicrobial mouthwashes during the last three months. Investigators consecutively recruited patients daily based on the inclusion/exclusion criteria.

The commercial product HY used as adjunctive to SRP is formulated as a liquid or gel containing an aqueous mixture of hydroxybenzenesulfonic, hydroxymethoxybenzene, and sulfuric acids (HY brochure). This product received clearance from the U.S. Food and Drug Administration and European Regulatory Agency as an adjunctive medical device for periodontal treatment.

Patients received an initial complete full-mouth clinical examination (baseline) and at three months follow-up after completing the subgingival instrumentation. After the initial clinical periodontal assessment and diagnosis, a standard, stepwise treatment plan was provided for each patient based on the available recommendations [11,28,29], which included the first-step therapy comprising of patient behavioral change interventions, supragingival biofilm control, and management of risk factors. Cause-related therapy consisting of mechanical subgingival instrumentation was initiated only after the first-step therapy endpoints were achieved.

For each periodontitis patient, two hemiarches of the four hemiarches were treated with SRP plus HY and were included in the test group (SRP plus HY group), while the other two hemiarches that received SRP alone were included in the control group (SRP group). After completing the study, patients received the other therapy steps as planned.

The hemiarches (hemiarches 1 and 4) were randomly assigned to one of the therapies (SRP plus HY or SRP) based on a randomization table [30]. On the first treatment day, hemiarches 1 and 4 were treated following random allocation (test therapy or control therapy); the following day, the other treatment was applied for the other two hemiarches (hemiarches 2 and 3). The records of the participants contained no mention of treatment allocation. The list containing allocation numbers and names of patients was kept by the senior periodontologist, who also recruited the patients (A.R.). The baseline examination and the treatment were provided by two investigators (A.S. and A.C.), and re-evaluations were performed by other investigators (I.C.M. and A.G.D.). The investigators conducting the follow-up examination and the statistician were unaware of the treatment type received.

### 2.2. Clinical Periodontal Examination

The investigators were previously calibrated at the initiation of another study [31]. At the initiation of the present study, the investigators received written instructions on the study protocol and participated in two training meetings coordinated by a senior periodontist (D.R.). The full-mouth periodontal examinations, excluding third molars, were performed in standard conditions with standard equipment (dental mirror and 1 mm marking periodontal probe (UNC-15 periodontal probe, Hu-Friedy, Chicago, IL, USA)). The probing depth (PD), gingival recession (GR), and clinical attachment loss (CAL) were evaluated at six sites per tooth, following the standard clinical definitions [32]. All probing measurements were rounded down to the nearest millimeter. The full-mouth Gingival Bleeding Index (GBI) [33] and Oral Hygiene Index (IHI) scores [34] were calculated as a percentage of the total number of sites with gingival bleeding upon probing or dental plaque divided by the total number of examined sites (four sites for each tooth).

### 2.3. SRP

Patients received SRP based on the full-mouth scaling approach in a temporary sequence, as described in the study design section. SRP was performed under local anesthesia first using ultrasonic instruments (Unit-P5 Booster Suprasson-Satelec, Acteon, Mount Laurel, NJ, USA), followed by manual instrumentation with Gracey curettes (Hu-Friedy, Chicago, IL, USA) for the final root planing. 

For the test group, just before scaling, the semi-viscous HY solution was topically applied into the periodontal pockets with an irrigation syringe with a blunt tip starting from the deepest portion and moving towards the more superficial areas. The product was applied for 20 s and then removed by thorough irrigation utilizing the ultrasonic device under strict evacuation with high-speed aspiration, as recommended by the producer. 

After treatment, all patients received at-home post-therapy care instructions. Patients were instructed to avoid antiplaque and anti-inflammatory mouthwashes or antibiotic therapy for the following six weeks after treatment.

### 2.4. Study Outcomes

The primary outcome was the difference in change in the total probing depth (PD) between the intervention groups. The secondary outcomes were the difference in intragroup changes and between the intervention groups in the total/interproximal probing depth, total/interproximal attachment loss, count of total/interproximal probing depths = 5 mm, and count of total/interproximal probing depths ≥ 6 mm, as well as the intragroup changes in the IHI and GBI scores.

### 2.5. Statistical Analysis

For each of the test (SRP plus HY) and control (SRP) groups, 60 quadrants were analyzed. The qualitative data were presented as absolute and relative frequencies. Normally distributed quantitative data were presented as means and standard deviations, while non-normally distributed data were presented as medians and interquartile ranges. The method of analyzing the results was intention-to-treat. Comparisons between baseline and follow-up, as well as comparisons for dependent samples for normally distributed data, were performed with a *t*-test for dependent samples and Wilcoxon signed-rank test for data not following the normal distribution. For all outcomes, a superiority hypothesis was used. The means of the differences between observations were presented, along with 95% confidence intervals. For all statistical tests, a 0.05 level of significance was used, as well as a two-tailed *p*-value. No corrections for multiple testing were employed. All analyses were carried out with the R environment for statistical computing and graphics (R Foundation for Statistical Computing, Vienna, Austria), version 4.1.2 [35].

## 3. Results

**Figure 1:**
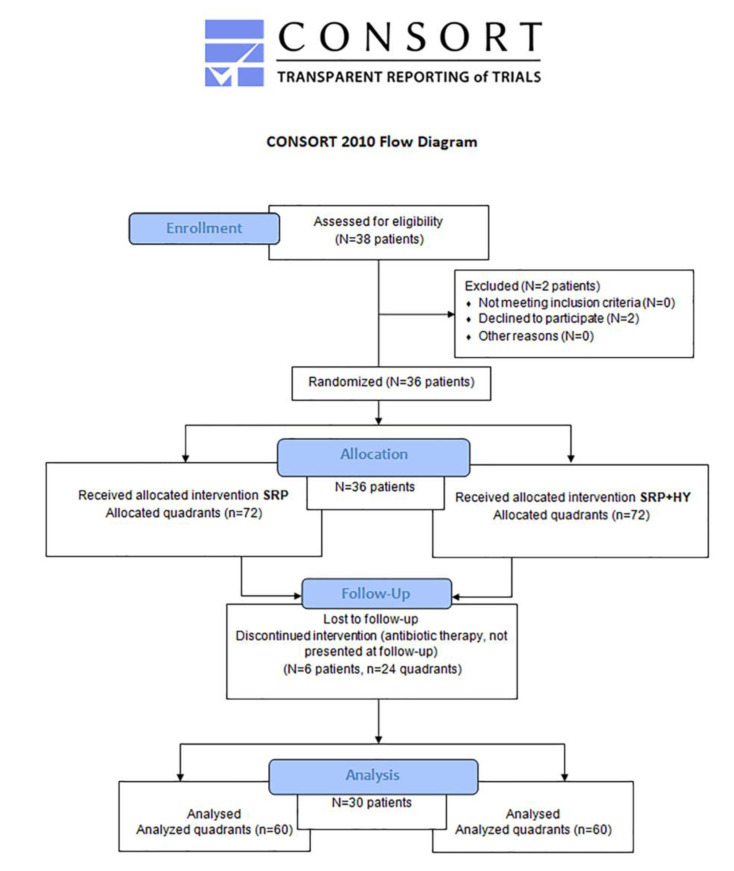
Flow chart of patients’ recruitment. Abbreviations: N and n, number; SRP, subgingival mechanical instrumentation; HY, HYBENX.

Of the patients with stages III and IV periodontitis meeting the inclusion criteria, thirty patients, with ages ranging from 29 to 72 years, completed the study (Figure 1). A total number of 719 teeth, corresponding to 4314 sites (of which 2876 proximal sites), were examined. There were very few missing data.

The demographic and clinical baseline characteristics of the investigated patients are summarized in Table 1.

Gingival tissues healed uneventfully in both treatment groups. At three months follow-up, the IHI index score (mean (SD) 42.24 (28.47–59.75)) and GBI index score (mean (SD) 31.99 (19.84–50.38)) indicated a statistically significant reduction (*p* < 0.0001) in the investigated patients.

Intragroup tests evaluating the evolution of periodontal pockets over time showed for both the test and control groups that the mean PD values resulting from all the examined sites (PDt), as well as from interproximal sites (PDip), were reduced at the three months follow-up as compared to baseline (Table 2). For both the control and test groups, statistically significant mean differences for both items were recorded (∆PDt_Baseline-Follow-up_ and ∆PDip_Baseline-Follow-up_, respectively) (*p* < 0.001). For the test and control sites, the same evolution pattern was recorded for the total and interproximal CAL values (CALt and CALip), with a statistically significant reduction of CALt and CALip at the follow-up compared to baseline (∆CALt_Baseline-Follow-up_ and ∆CALip_Baseline-Follow-up_, *p* < 0.001, *p* = 0.003).

The intergroup comparisons of each mean value of PDt_Follow-up_, PDip_Follow-up_, CALt_Follow-up_, CALip_Follow-up_, ∆PDt_Baseline-Follow-up_, ∆PDip_Baseline-Follow-up_, ∆CALt_Baseline-Follow-up_, and ∆CALip_Baseline-Follow-up_ in the test group to the corresponding values in the control group showed no significant differences (*p* = 0.322, *p* = 0.36, *p* = 0.516, *p* = 0.509, *p* = 0.674, *p* = 0.487, *p* = 0.821, and *p* = 0.821, respectively).

A statistically significant reduction of the number of pockets with PD = 5 mm after treatment as compared to the baseline was calculated for both treatment groups and for both the total sites and interproximal sites (count ∆PDt_Baseline-Follow-up_ and count ∆PDip_Baseline-Follow-up_) (*p* < 0.001) (Table 3). For the control group, the calculated percentages of closed pockets (transformation of 5 mm pocket depths into normal values of 4 mm maximum) for PDt and for PDip were 40% and 35.2%, respectively. For the test group, the percentages of closed pockets for PDt and PDip were 46% and 46.5%, respectively. 

For both treatment groups, a more important reduction in the number of deep pockets (count ∆PDt_Baseline-Follow-up_ and count ∆PDip_Baseline-Follow-up_) of ≥6 mm was recorded (*p* < 0.001) (Table 3). For the test group, the percentages of transformation from initially deep pockets (PD ≥ 6 mm) into shallower ones were 88.4% for the total sites (PDt) and 68.8% for the interproximal sites (PDip). For the test group, the corresponding percentages were 60.6% and 59%, respectively.

Intergroup comparisons of the number of deep pockets (PD ≥ 6 mm) revealed significant differences in the count of total sites PDt_Follow-up_ (*p* < 0.001) and count of total sites ∆PDt_Baseline-Follow-up_ (*p* = 0.005) (Table 3). 

Table 4 shows statistically significantly higher mean interproximal pocket depth values than the total pocket depth values at baseline, as well as at the follow-up for both treatment groups.

## 4. Discussion

This split-mouth RCT evaluated the potential additive clinical benefit of a local irrigant with antiseptic and desiccation properties (HY) in conjunction with SRP as compared to the therapeutical gold standard represented by SRP. 

After three months, no significant differences in the clinical periodontal parameters between the test and control treatment groups were recorded, which supports our original study hypothesis. No additional PD reduction could be attributed to the HY application for either total or interproximal periodontal pockets. Our results are in agreement with data from other similar studies. Thus, a split-mouth RCT reported no remarkable differences in the PD reduction between sites receiving combined therapy (SRP plus HY) and those receiving only mechanical instrumentation (SRP) (4.69 ± 1.7 mm vs. 4.95 ± 1.7 mm, *p* = 0.04) [24].

The lack of additive effect of HY may be due to the relatively short contact of the product with the subgingival structures. The reapplication of the product after SRP to augment its antimicrobial efficacy could be considered for future evaluations. A marked reduction of the subgingival bacterial burden is a key element targeted during periodontitis non-surgical therapy in order to reduce inflammation and obtain proper periodontal healing and stability [22,36]. 

A study with a similar design and similar primary and secondary outcomes reported opposite results [22]. This split-mouth RCT showed a statistically significant PD reduction (3.25 ± 0.57 mm vs. 2.23 ± 0.31 mm, *p* < 0.05) and clinical attachment level modification (4.21 ± 0.34 mm vs. 3.16 ± 0.29 mm, *p* < 0.001) in sites receiving combined treatment (SRP plus HY) compared to sites receiving only SRP.

In a parallel randomized controlled trial, deep periodontal pockets in teeth deemed hopeless were treated with either SRP plus HY or only SRP [37]. The evaluation after extraction showed a significantly reduced percentage of the root surface (*p* < 0.001) still covered with soft debris on those teeth receiving combined therapy (10.63 ± 12.79%) as opposed to those receiving single therapy (17.32 ± 10.68%). On the contrary, a significantly higher percentage of the root surface covered by hard deposits (*p* < 0.001) was observed on teeth surfaces treated with the combined approach (46.90 ± 33.76%) as compared with the single approach (23.65 ± 18.27%). A significantly increased penetration of the cleaning depth was observed in those teeth receiving the combined therapy (4.41 ± 2.96 mm) as opposed to teeth receiving a single therapy (2.67 ± 2.70 mm). These results indicate that HY does not facilitate the calculus detachment from the treated root surfaces but only plaque disruption [18]. Thus, this product containing sulphonic/sulphuric acids can favor the active detachment of biofilms from the infected subgingival areas through its rapid desiccation capacity facilitating the removal of the biofilm using mechanical instruments [22,24]. 

On the other hand, in our study, both the test (SRP plus HY) and control (SRP) treatments determined a statistically significant improvement of the periodontal parameters at the follow-up visit as compared to baseline, as has been proven by numerous systematic [38,39,40] and narrative [41,42] reviews. In the treatment (SRP plus HY) and control (SRP) groups, the mean PD reductions were 0.82 mm and 0.85 mm, respectively, when considering all PD values (PDt) and 0.96 mm and 1.01 mm, respectively, when considering only interproximal PD (PDip). The current evidence revealed a mean PD reduction of 1.7 mm at 6–8 months after SRP, with greater values of 2.6 mm for the initially deep pockets (>6 mm) [11].

Regarding the evolution in the number (count) of periodontal pockets, our study found that 40% of the total and 35.2% of the interproximal sites with pocket depths of 5 mm closed after the control treatment (SRP). Following the test treatment (SRP plus HY), 46% of the total and 46.46% of the interproximal sites with pocket depths of 5 mm closed. These represented the pockets that no longer required further treatment. Higher reduction percentages in the number of deep pockets (PD ≥ 6 mm) were recorded for both the total and interproximal number of pockets and for both the control group (88.4% and 68.8%, respectively) and test group (60.6% and 59%, respectively). According to the scientific literature, subgingival instrumentation resulted in a mean proportion of closed pockets of 74% [11]. 

The combined treatment (SRP plus HY) and the single therapy (SRP) determined a statistically significant mean clinical attachment gain of 0.35 mm and 0.3 mm, respectively, when all the sites were included and 0.36 mm and 0.33 mm, respectively, when only the interproximal sites were considered, which was less than the literature reported [11]. It is important to emphasize that this RCT encompassed cases with severe destruction and a variety of susceptibility factors that posed a difficult challenge for achieving ideal treatment outcomes.

One positive aspect in our study was the differentiated assessment of the total PD and interproximal PD, since the periodontal breakdown is usually more severe and more frequently located in the interproximal areas associated with more biofilm accumulation and persistence. Adding buccal and oral PD values, which are usually less deep than interproximal PD measurements, could have diluted the overall mean PD values, making the pathological changes less obvious. The mean PDip baseline values were significantly higher than the mean PDt values for both the control and test groups (4.54 (0.74) mm vs. 3.87 (0.66) mm, *p* < 0.001 and 4.58 (0.76) mm vs. 3.94 (0.67) mm, *p* = 0.001, respectively). The same pattern was recorded at the follow-up (3.53 (0.7) mm vs. 3.02 (0.67) mm, *p* = 0.005 and 3.62 (0.8) mm vs. 3.12 (0.69) mm, *p* = 0.011, respectively).

The significant reduction of Bleeding Index scores for both treatment groups was highlighted by an improved Oral Hygiene Index, as expected. Although the Bleeding Index score was reduced by almost 50%, its follow-up value was far from supporting periodontal stability. This might be due to, on the one hand, the plaque index that was higher than the recommended threshold value and, on the other hand, the severely treated forms of periodontitis that were more difficult to manage therapeutically [43].

HY was selected in our study due to the serious concerns related to the increased bacterial resistance associated with systemic antibiotic therapy, which indicates the need for collective efforts to identify alternative adjunctive products that can enhance the antimicrobial effect of subgingival instrumentation [22,44]. However, HY has received less attention in research, and its mechanism of action remains unclear [18]. 

No remarkable side effects were reported by participants in this study, except for dentin hypersensitivity for both treatment groups, which is explainable, since deep pockets are associated with large root surface exposure. Other authors have reported more pain or post-operative dentin hypersensitivity in groups treated with HY plus SRP than in groups treated with SRP only [36]. Marked gingival recessions have also been reported after HY applications in periodontal abscesses [20].

Although some concerns have been raised about the possible cytotoxicity related to the product’s composition, HY applications were generally well tolerated and associated with good gingival healing in our group of patients. There is scarce information with respect to HY effects on the cellular level, and the currentlyavailable data are inconsistent. Some authors have reported that HY might have no, or only limited, cytotoxicity on MG-63 osteoblast-like cells [45]. In another study, HY inhibited, in a dose-dependent manner, the proliferation of human gingival mesenchymal stromal cells but did not significantly affect the cells’ viability, regardless of its concentrations [46].

The lack of quantification of the secondary effects and microbiological monitoring might be considered a limitation of the present study. However, due to the variable spread of bacteria across different oral sites, the consistency of the microbiological assessments in split-mouth studies might be questionable.

Further extensive research is required to determine the potential benefits of HY in periodontitis therapy since the available studies on the effectiveness of this product are limited and have yielded conflicting results.

## 5. Conclusions

Both therapeutic approaches resulted in statistically significant improvements in the monitored periodontal parameters. This study did not identify any additional benefits from HY subgingival applications when used in conjunction with subgingival instrumentation. The evolution of the interproximal probing depths could be an alternative to reporting periodontitis treatment outcomes rather than considering the total pocket depths, as most periodontal destruction tends to occur predominantly at interproximal sites. 

## Figures and Tables

**Table 1:** Demographic and clinical baseline characteristics.

Characteristics	Mean (SD)/ Median (IQR) (N = 30)	Mean (SD)/ Median (IQR) SRP (n = 60)	Mean (SD)/ Median (IQR) SRP + HY (n = 60)
Age	44.8 (10.76)		
Gender (M vs. F)	17/30 (56.67)		
IHI score % _Baseline_	80 (63.77–100)		
GBI score % _Baseline_	60.43 (42–83.17)		
Total sites PDt _Baseline_	3.90 (0.66)	3.87 (0.66)	3.94 (0.67)
Interproximal sites PDip _Baseline_	4.56 (0.75)	4.54 (0.74)	4.58 (0.76)
Count total sites PDt _Baseline_ = 5 mm	12.38 (7.76)	13.27 (9.06)	11.5 (6.46)
Count interproximal sites PDip _Baseline_ = 5 mm	10.38 (6.18)	10.87 (6.81)	9.9 (5.55)
Count total sites PDt _Baseline_ ≥ 6 mm	13.32 (9.28)	13.27 (9.06)	13.37 (9.51)
Count interproximal sites PDip _Baseline_ ≥ 6 mm	12.25 (8.39)	12.3 (8.03)	12.2 (8.76)
Total sites CALt _Baseline_	4.32 (1.54)	4.26 (1.54)	4.39 (1.54)
Interproximal sites CALip _Baseline_	4.79 (1.66)	4.73 (1.66)	4.85 (1.67)

Abbreviations: F, female; CAL, clinical attachment loss; IHI, Oral Hygiene Index; IQR, interquartile range; ip, interproximal; GBI, Gingival Bleeding Index; HY, HYBENX; M, male; N, number of patients; n, number of quadrants; SD, standard deviation; SRP, subgingival mechanical instrumentation; t, total.

**Table 2:** Evolution of the probing depths and clinical attachment levels after treatment.

Group	Control SRP (n = 60)	Test SRP + HY (n = 60)	Difference (95% CI)	*p*-Value
**Total probing depths (PDt) (mm) (6 values/tooth)**				
Total sites PDt _Baseline_, Mean (SD)	3.87 (0.66)	3.94 (0.67)	−0.07 (−0.23–0.09)	0.379
Total sites PDt _Follow-up_, Mean (SD)	3.02 (0.67)	3.12 (0.69)	−0.1 (−0.29–0.1)	0.322
Total sites ∆ PDt _Baseline-Follow-up_, Mean (95% CI)	0.85 (0.68–1.02)	0.82 (0.69–0.96)	0.03 (−0.1–0.15)	0.674
***p*-Value**	<0.001	<0.001		
**Interproximal probing depths (PDip) (mm) (4 values/tooth)**				
Interproximal sites PDip _Baseline_, Mean (SD)	4.54 (0.74)	4.58 (0.76)	−0.04 (−0.23–0.15)	0.691
Interproximal sites PDip _Follow-up_, Mean (SD)	3.53 (0.7)	3.62 (0.8)	−0.09 (−0.28–0.11)	0.36
Interproximal sites ∆ PDip _Baseline-Follow-up_, Mean (95% CI)	1.01 (0.8–1.22)	0.96 (0.79–1.13)	0.05 (−0.09–0.19)	0.487
***p*-Value**	<0.001	<0.001		
**Total clinical attachment loss (CALt) (mm) (6 values/tooth)**				
Total sites CALt _Baseline_, Mean (SD)	4.26 (1.54)	4.39 (1.54)	−0.13 (−0.39–0.12)	0.292
Total sites CALt _Follow-up_, Mean (SD)	3.96 (1.4)	4.04 (1.47)	−0.08 (−0.34–0.18)	0.516
Total sites ∆ CALt _Baseline-Follow-up_, Mean (95% CI)	0.3 (0.15–0.45)	0.35 (0.16–0.55)	−0.02 (−0.22–0.18)	0.821
***p*-Value**	<0.001	<0.001		
**Total interproximal attachment loss values (CALip) (mm) (4 values/tooth)**				
Interproximal sites CALip _Baseline_, Mean (SD)	4.73 (1.66)	4.85 (1.67)	−0.12 (−0.43–0.2)	0.447
Interproximal sites CALip _Follow-up_, Mean (SD)	4.39 (1.52)	4.49 (1.57)	−0.1 (−0.39–0.2)	0.509
Interproximal sites ∆ CALip _Baseline-Follow-up_, Mean (95% CI)	0.33 (0.17–0.5)	0.36 (0.13–0.58)	−0.02 (−0.22–0.18)	0.821
***p*-Value**	<0.001	0.003		

Abbreviations: CAL, clinical attachment loss; CI, confidence interval; ip, interproximal; HY, HYBENX; PD, probing depth; SD, standard deviation; SRP, subgingival mechanical instrumentation; t, total; ∆, difference.

**Table 3:** Changes in the number of periodontal pockets after treatment.

Group	Control SRP (n = 60)	Test SRP + HY (n = 60)	Difference (95% CI)	*p*-Value
**Mean count of total sites with PD = 5 mm (PDt) (6 values/tooth)**				
Count total sites PDt _Baseline_, Mean (SD)	13.27 (9.06)	11.5 (6.46)	1.77 (1.59–5.12)	0.291
Count total sites PDt _Follow-up_, Mean (SD)	7.9 (5.92)	6.2 (5.47)	1.7 (−0.45–3.85)	0.116
Count Total sites ∆ PDt _Baseline-Follow-up_, Mean (95% CI) ***p*-Value**	5.37 (2.31–8.42) <0.001	5.3 (3.23–7.37) <0.001	0.07 (−4.04–4.18)	0.974
**Mean count of interproximal sites with PD = 5 mm (PDip) (4 values/tooth)**				
Count interproximal sites PDip _Baseline_, Mean (SD)	10.87 (6.81)	9.9 (5.55)	0.97 (−1.18–3.12)	0.365
Count interproximal sites PDip _Follow-up_, Mean (SD)	7.03 (5.03)	5.3 (4.4)	1.73 (−0.25–3.72)	0.084
Count interproximal sites ∆ PDt _Baseline-Follow-up_, Mean (95% CI) ***p*-Value**	3.84 (1.67–5.99) <0.001	4.6 (2.63–6.57) <0.001	−0.77 (−2.81–1.28)	0.449
**Mean count of total sites with PD ≥ 6 mm (PDt) (6 values/tooth)**				
Count total sites PDt _Baseline_, Mean (SD)	13.27 (9.06)	13.37 (9.51)	−0.1 (−2.47–2.27)	0.932
Count total sites PDt _Follow-up_, Mean (SD)	1.53 (2.49)	5.27 (5.91)	−3.73 (−5.59–−1.88)	< 0.001
Count Total sites ∆ PDt _Baseline-Follow-up_, Mean (95% CI) ***p*-Value**	11.73 (8.88–14.59) <0.001	8.1 (5.58–10.62) <0.001	3.63 (1.16–6.11)	0.005
**Mean count of interproximal sites with PD ≥ 6 mm (PDip) (4 values/tooth)**				
Count interproximal sites PDip _Baseline_, Mean (SD)	12.3 (8.03)	12.2 (8.76)	0.1 (−2.04–2.24)	0.925
Count interproximal sites PDip _Follow-up_, Mean (SD)	3.83 (5.28)	5 (5.63)	−1.17 (−2.63–0.3)	0.115
Count interproximal sites ∆ PDip _Baseline-Follow-up_, Mean (95% CI) ***p*-Value**	8.47 (6.06–10.88) <0.001	7.2 (4.89–9.51) <0.001	1.27 (0.98–3.52)	0.259

Abbreviations: CI, confidence interval; ip, interproximal; HY, HYBENX; PD, probing depth; SD, standard deviation; SRP, subgingival mechanical instrumentation; t, total; ∆, difference.

**Table 4:** The comparison of the mean pocket depth values of the interproximal sites and total sites.

	Mean Interproximal PDip (mm)	Mean Total PDt (mm)	Difference (95% CI)	*p*-Value
	SRP (n = 60)	SRP (n = 60)		
Baseline, Mean (SD)	4.54 (0.74)	3.87 (0.66)	0.67 (0.31–1.04)	<0.001
Follow-up, Mean (SD)	3.53 (0.7)	3.02 (0.67)	0.51 (0.16–0.87)	0.005
	**SRP + HY** **(n = 60)**	**SRP + HY** **(n = 60)**		
Baseline, Mean (SD)	4.58 (0.76)	3.94 (0.67)	0.64 (0.27–1.01)	0.001
Follow-up, Mean (SD)	3.62 (0.8)	3.12 (0.69)	0.51 (0.12–0.89)	0.011

Abbreviations: CI, confidence interval; HY, HYBENX; PD, probing depth; SD, standard deviation; SRP, subgingival mechanical instrumentation.

## Data Availability

The datasets generated during the current study are available from the corresponding author upon reasonable request.
